# Depression and anxiety in empty nose syndrome: A systematic review and Meta-analysis

**DOI:** 10.1007/s00405-025-09535-1

**Published:** 2025-07-05

**Authors:** Anuja H. Shah, Isabelle J. Chau, Shaun A. Nguyen, Alexander N. Duffy, Zachary M. Soler, Rodney J. Schlosser

**Affiliations:** 1 Department of Otolaryngology – Head and Neck Surgery, Medical University of South Carolina, Charleston, 135 Rutledge Ave, 29425 SC USA; 2 Georgetown University School of Medicine, Washington, DC USA; 3 New York Medical College School of Medicine, Valhalla, NY USA

**Keywords:** Empty nose syndrome, Atrophic rhinitis, Depression, Anxiety, Mental health

## Abstract

**Background:**

Empty nose syndrome (ENS) is a condition characterized by a persistent feeling of nasal obstruction despite having a patent nasal airway. This study aims to understand prevalence of depression and anxiety in patients with ENS and the impact of augmentation procedures on psychological symptoms.

**Methods:**

The literature was searched in accordance with PRISMA guidelines. Outcome measures were collected for mental health prevalences and patient-related outcome measures (PROMs).

**Results:**

Of 1056 abstracts identified, 11 studies (*N* = 401 patients) were included. Among patients with ENS, prevalences of depression and anxiety were 76.6% (95%CI: 61.0-89.1) and 77.0% (95% CI: 49.9–95.5). The prevalence of depression in patients with ENS was significantly increased compared to that of patients with chronic rhinosinusitis (CRS) (RR: 2.8 [95%CI: 1.5–5.2], *p* = 0.001) in studies which compared both conditions. In patients who underwent augmentation, Patient Health Questionnaire-9 and Generalized Anxiety Disorder-7 showed no significant improvement at 6-month follow-up (5.39 [95%CI: -0.7-11.5], *p* = 0.08; 5.45 [95%CI: -2.8-13.7], *p* = 0.20) while Beck Depression Inventory-II and Beck Anxiety Inventory showed significant improvement at 6-month follow-up (11.94 [95%CI: 8.7–15.2], *p* < 0.00001; 10.83 [95%CI: 7.7–14.], *p* < 0.0001).

**Conclusions:**

Depression and anxiety are common among patients with ENS, with a possible increased prevalence of depression in patients with ENS compared to those with CRS. Patients with ENS demonstrated inconsistent improvement in depression and anxiety scores following augmentation procedures depending on the PROMs utilized. As patients with ENS often have mental health comorbidities, further research should investigate whether anxiety and depression are true sequelae of ENS or factors contributing to its development.

**Supplementary Information:**

The online version contains supplementary material available at 10.1007/s00405-025-09535-1.

## Introduction

Empty Nose Syndrome (ENS) is a rare, iatrogenic condition characterized by perception of paradoxical nasal obstruction despite a patent nasal passage. ENS is associated with the over-resection of the nasal turbinates during surgery, most commonly the inferior turbinate, leading to symptoms of nasal congestion, dryness, crusting, burning, dyspnea, sensations of abnormal airflow, and hypersensitivity to cold air [1–3]. It is difficult to determine the prevalence of ENS as symptoms may not present for many months to years following initial iatrogenic insult; however, several studies have reported a rate of 8–22% of patients developing ENS following turbinate resection [2]. Current non-surgical interventions in the management of ENS include nasal irrigation methods to address dryness and specialized nasal plugs to address abnormal airflow [1, 3, 4]. Procedural interventions include augmentation with bio-injectable materials or submucosal implantation of various materials including cartilage [1, 5, 6].

In addition to rhinologic symptoms, patients with ENS often experience psychiatric symptoms, including chronic fatigue, frustration, irritability, anger, anxiety, depression, and suicide [7]. The pathophysiology of mental status changes in patients with ENS is not well understood, but the increased prevalence of mental health disorders in patients with ENS has been noted [8–11]. The psychological impact is often underappreciated in both diagnosis and treatment. In determining treatment, surgical providers may focus primarily on the physical or structural aspects of the condition, but may not consider the psychological factors, such as anxiety, depression, or the stress of dealing with a rare and poorly understood condition [12].

The aim of this study is to systematically review the literature and perform a meta-analysis on the prevalence of depression and anxiety in patients with ENS as well as the impact of augmentation procedures on psychological symptoms. A comprehensive understanding of the prevalences of anxiety and depression and the impact of augmentation in patients with ENS will allow for clinicians to determine the best course of management for the treatment of both rhinologic and psychiatric symptoms.

## Methods and materials

### Research question

The question guiding this systematic review is “What is the prevalence of depression and anxiety in patients with empty nose syndrome and do augmentation procedures impact depression and anxiety?” We hypothesize that patients with ENS have an increased risk for co-morbid depression and anxiety. The subjects of interest were patients with a diagnosis of empty nose syndrome. The outcomes we reported were depression and anxiety in patients with ENS and chronic rhinosinusitis (CRS) if reported as a comparison group to ENS, as well as patient-reported outcome measures (PROMs) prior to and following augmentation procedures.

### Search criteria

This systematic review was conducted in accordance with the preferred reporting items for systematic reviews and meta-analyses (PRISMA) [13]. A literature search was conducted using PubMed (National Library of Medicine), Scopus (Elsevier), CINAHL (EBSCO), Cochrane Library, and PsycINFO. A literature search designed for included keywords and medical subjected headings (MeSH) related to empty nose syndrome, paradoxical nasal obstruction, secondary atrophic rhinitis, depression, anxiety, and mental health. Once the literature search was completed, articles were uploaded to Covidence systematic review software (Veritas Health Innovation Ltd. Melbourne, Australia).

### Study selection

Two authors (A.H.S and I.J.C.) independently screened articles for inclusion by title and abstract first, followed by full text review. Discrepancies between reviewers were resolved via discussion by the two reviewers. Inclusion criteria were (1) patients with a diagnosis of empty nose syndrome and (2) inclusion of data on the prevalence or severity of mental health conditions. Exclusion criteria were (1) animal studies, (2) case studies, (3) review articles, (4) articles with non-extractable data, (5) studies without available English translation. For studies in which the institution, time period, and inclusion criteria for patients overlapped, the study cohort with the most recent publication date or largest sample size was selected to avoid inflation of effect estimates.

### Study appraisal

Each study was assigned a level of evidence according to the Oxford Center for Evidence-Based Medicine criteria [14]. Nonrandomized control trials were assessed for risk of bias with the Risk of Bias in Non-Randomized Studies – of Exposures (ROBINS-E) assessment tool [15]. Cross-sectional studies were assessed for risk of bias using the Joanna Briggs Institute (JBI) Critical Appraisal Tool checklist [16]. Two authors (A.H.S and I.J.C.) independently assessed each study using a checklist, and all conflicting answers were reconciled discussion by the two reviewers. For studies using the ROBINS-E assessment, each aspect of risk of bias was assigned a grade of low, unclear, or high. For studies in which the JBI was utilized, each item was given a score of “1” for “yes” and “0” for “no,” “not applicable,” or “unclear.” The cross-sectional checklist has 8 questions and a score of 4 or higher was considered at low risk for bias.

### Data extraction

Two authors (A.H.S and I.J.C.) independently extracted data into a standardized spreadsheet. Studies which did not provide raw data upon request were extracted from figures using computer-vision based tool, PlotDigitizer version 4.8 (Porbital 2025). Any conflicts or inaccuracies were resolved via discussion by the two reviewers. Extracted data included author name, year of publication, sample size, sample demographics, disease characteristics, treatment characteristics, and comorbidities in ENS patients. Outcome data included prevalence of depression and anxiety in ENS patients as determined by PROMs, as well as pre-augmentation and post-augmentation mental health-related PROM scores such as Patient Health Questionnaire-9 (PHQ-9), Patient Health Questionnaire-15 (PHQ-15), Generalized Anxiety Disorder-7 (GAD-7), Beck Depression Inventory-II (BDI-II), Beck Anxiety Inventory (BAI), Empty Nose Syndrome 6-Item Questionnaire (ENS6Q), and Sinonasal Outcome Test-22 and − 25 (SNOT22 and SNOT25). For the purposes of this study, augmentation was considered either implantation or injection. In studies comparing the prevalence of depression in ENS and CRS patients, outcomes regarding CRS patients were also extracted.

### Statistical analysis

Meta-analysis of continuous measures (age) was performed with Comprehensive Meta-Analysis version 4 (Biostat Inc, Englewood, NJ, USA). Meta-Analysis mean difference/standardized mean difference (pre-augmentation vs. post-augmentation) for depression, anxiety, and rhinologic symptom score outcome measures, and meta-analysis of risk ratio (ENS vs. CRS) for and depression was performed with Cochrane Review Manager (RevMan) version 5.4 (The Cochrane Collaboration 2020, United Kingdom). Meta-analysis of proportions (patient characteristics, prevalence, etc.) was performed using MedCalc 22.017 (MedCalc Software, Ostend, Belgium). Each measure (mean, proportion [%], mean difference [Δ], risk ratio [RR] and 95% confidence interval [CI]) was weighted according to the number of patients affected. Both the fixed effects model and the random effects model were used in this study. If there was high heterogeneity (I^2^ > 50%), then a random effects model was used; if low heterogeneity (I^2^ < 50%), then a fixed effects model is allowable [17, 18]. In addition, a comparison of proportions, expressed as difference (Δ) and 95% CI was done to compare outcomes between two groups. Sensitivity analyses were conducted to ensure findings were not driven by a few studies with overlapping data. Finally, potential publication bias was evaluated by visual inspection of the funnel plot and Egger’s regression test, which statistically examines the asymmetry of the funnel plot [19, 20]. A p-value of less than 0.05 was considered a statistically significant difference for all statistical tests.

## Results

### Search results and study characteristics

**Fig. 1:**
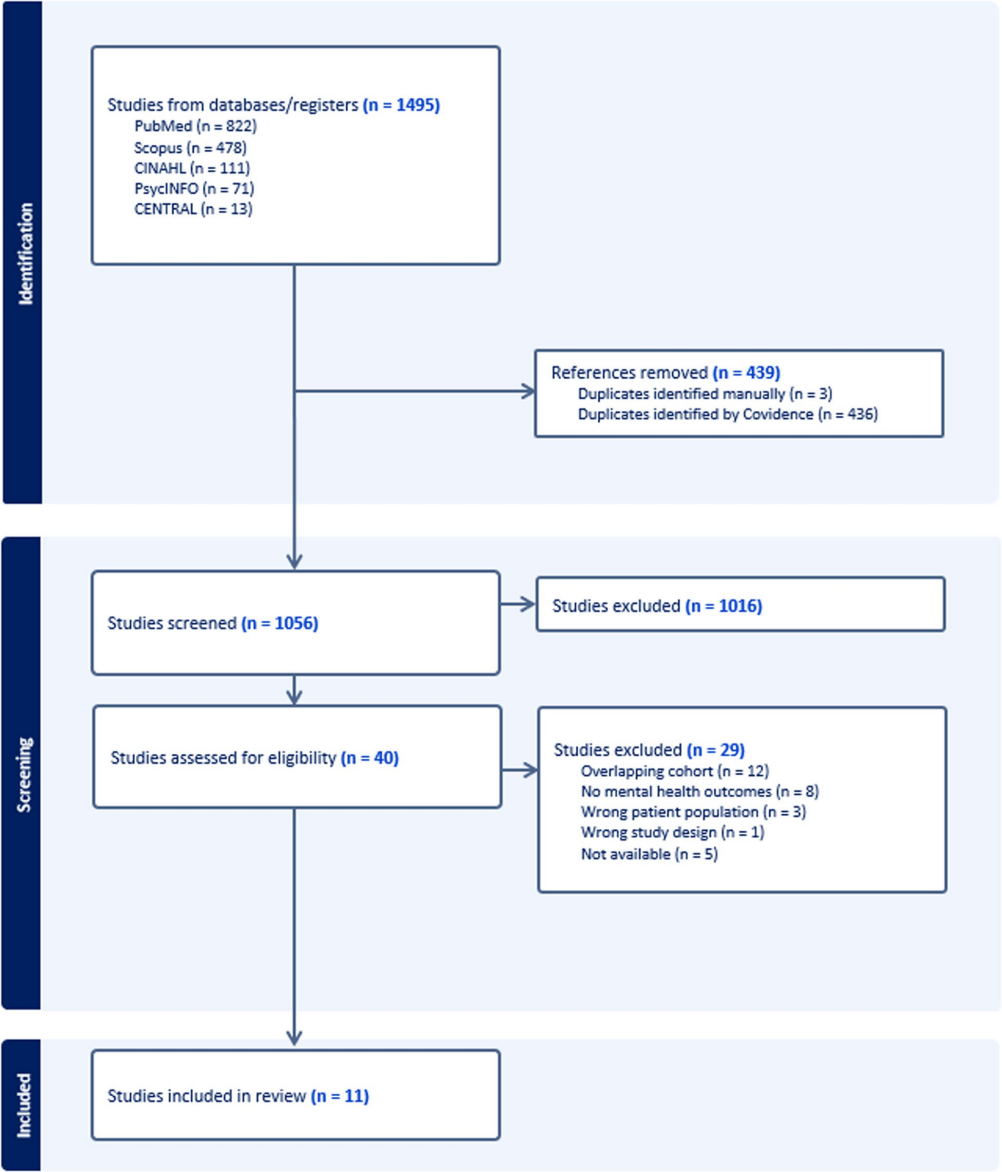
Preferred reporting items for systematic reviews and meta-analyses (PRISMA) flow diagram

The literature search yielded 1,495 studies, of which 439 duplicates were removed. The remaining 1,056 articles underwent title and abstract screening. Full-text review was performed on 40 studies and 29 studies were excluded. Reasons for exclusion included overlapping cohorts, no mental health outcomes, non-ENS patient population, improper study design, non-English text, and unavailable full text. At the conclusion of the review, 11 articles met the inclusion criteria [8, 10, 12, 21–28]. A PRISMA diagram detailing the systematic review is provided in Fig. 1. A summary of the studies included is provided in Table 1.

**Table 1:** Summary of included studies

Author Year	OLE	*N*	Empty Nose Diagnostic Criteria	Psychiatric Exclusion Criteria	Outcomes
Borchard 2019	4	14	History of turbinoplasty Evident IT tissue loss ENS6Q *≥* 11 Positive cotton test	None	PHQ-9, GAD-7, SNOT-22, ENS6Q
Dholakia 2021	4	17	History of IT reduction Evident IT tissue loss ENS6Q *≥* 11 Positive cotton test	None	PHQ-9, GAD-7, SNOT-22, ENS6Q
Hsueh 2023	4	35	History of turbinectomy Evident IT tissue loss ENS6Q *≥* 11 Positive cotton test	History of psychiatric disorders or use of antipsychotic medication prior to turbinate surgery	BDI-II, BAI, SNOT-25, ENS6Q, DAP
Huang 2021	4	54	Evidence of wide nasal airway Subjective symptoms Positive cotton test	Previously diagnosed with psychiatric disorders (schizophrenia, bipolar disorder, or major depression)	BDI-II, BAI, SNOT-25, ENS6Q, DAP
Huang 2024	4	74	History of IT procedure Evident IT tissue loss ENS6Q *≥* 11 Positive cotton test	Psychiatric disorders managed by psychiatrists	BDI-II, BAI, SNOT-25, ENS6Q
Kim 2021	4	24	History of previous nasal surgery Subjective symptoms Positive cotton test	Already diagnosed with depression before diagnosis of nasal disease	BDI-II, DAP
Lamb 2022	3	58	Diagnosis of ENS by a physician ENS6Q *≥* 11	None	PHQ-9, PHQ-15, ENS6Q, DAP
Lee 2016	4	20	Diagnosis of ENS History of middle/inferior turbinectomy Evidence of wide nasal cavity Positive cotton test	Any psychiatric disorders or received antipsychotics prior to study	BDI-II, BAI, DAP
Manji 2018	3	53	History of IT reduction Evident IT tissue loss ENS6Q *≥* 11	None	PHQ-9, GAD-7, ENS6, DAP
Thamboo 2020	4	10	History of IT reduction/resection Evidence of wide nasal cavity ENS6Q *≥* 11 Positive cotton test	None	PHQ-9, GAD-7, SNOT-22, ENS6Q
Tian 2021	4	28	History of nasal surgery Evidence of nearly normal nasal cavities/normal nasal function ENS6Q > 18; SNOT-25 > 57 Positive cotton test	Patients with any psychiatric disorders aside from Somatic Symptom Disorder	PHQ-9, GAD7, SNOT-25, PHQ-15, DAP

*Abbreviations: Oxford Level of Evidence (OLE)*,* Sample Size (N)*,* Inferior Turbinate (IT)*,* Patient Health Questionnaire − 9/−15 (PHQ-9/−15)*,* Generalized Anxiety Disorder-7 (GAD-7)*,* Beck Depression Inventory-II (BDI-II)*,* Beck Anxiety Inventory (BAI)*,* Sino-Nasal Outcome Test-22/−25 (SNOT-22/−25)*,* Empty Nose Syndrome 6-Item Questionnaire (ENS6Q)*,* Depression and/or Anxiety Prevalence (DAP)*

**Fig. 2:**
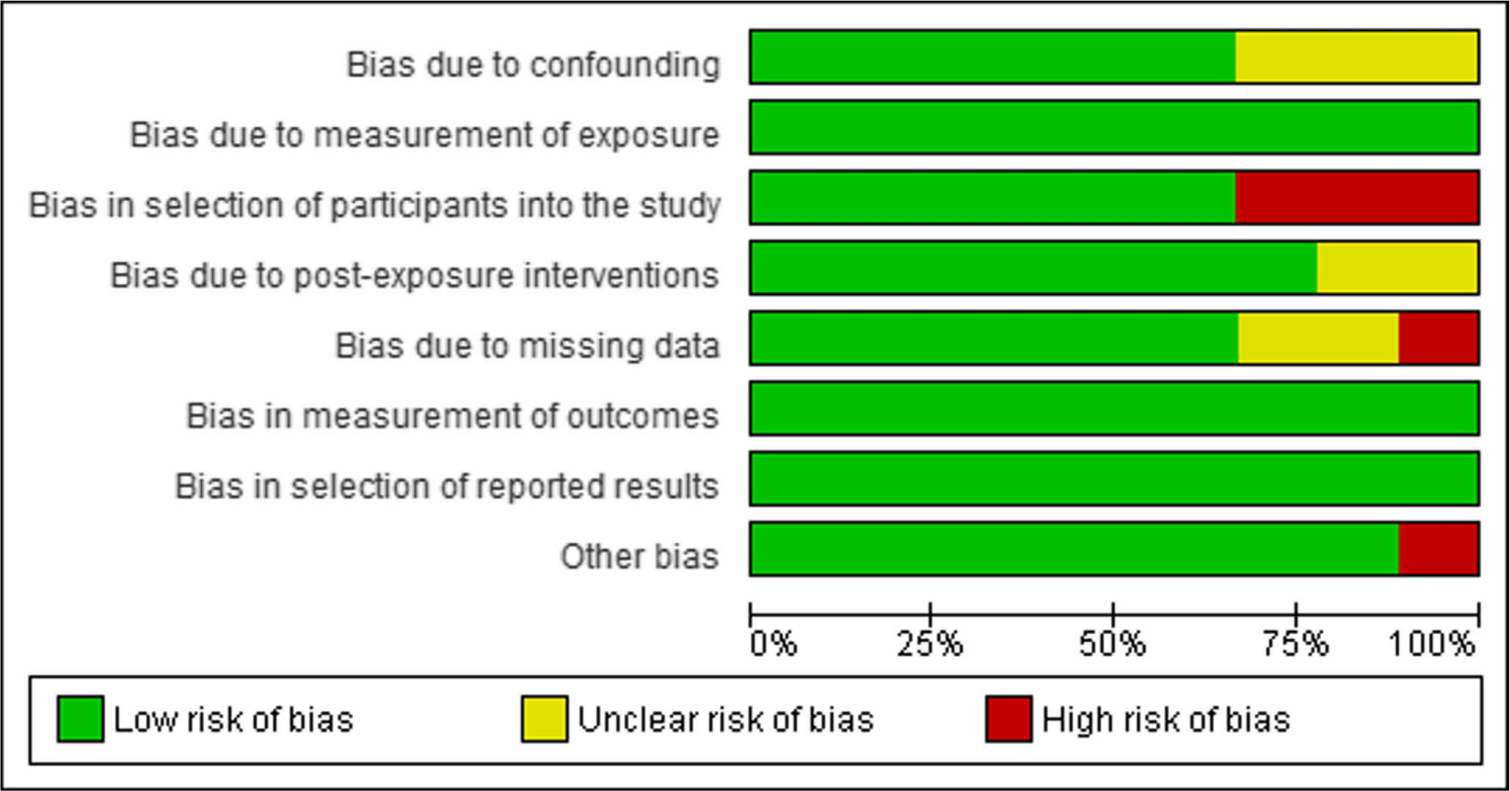
Risk of bias

**Fig. 3:**
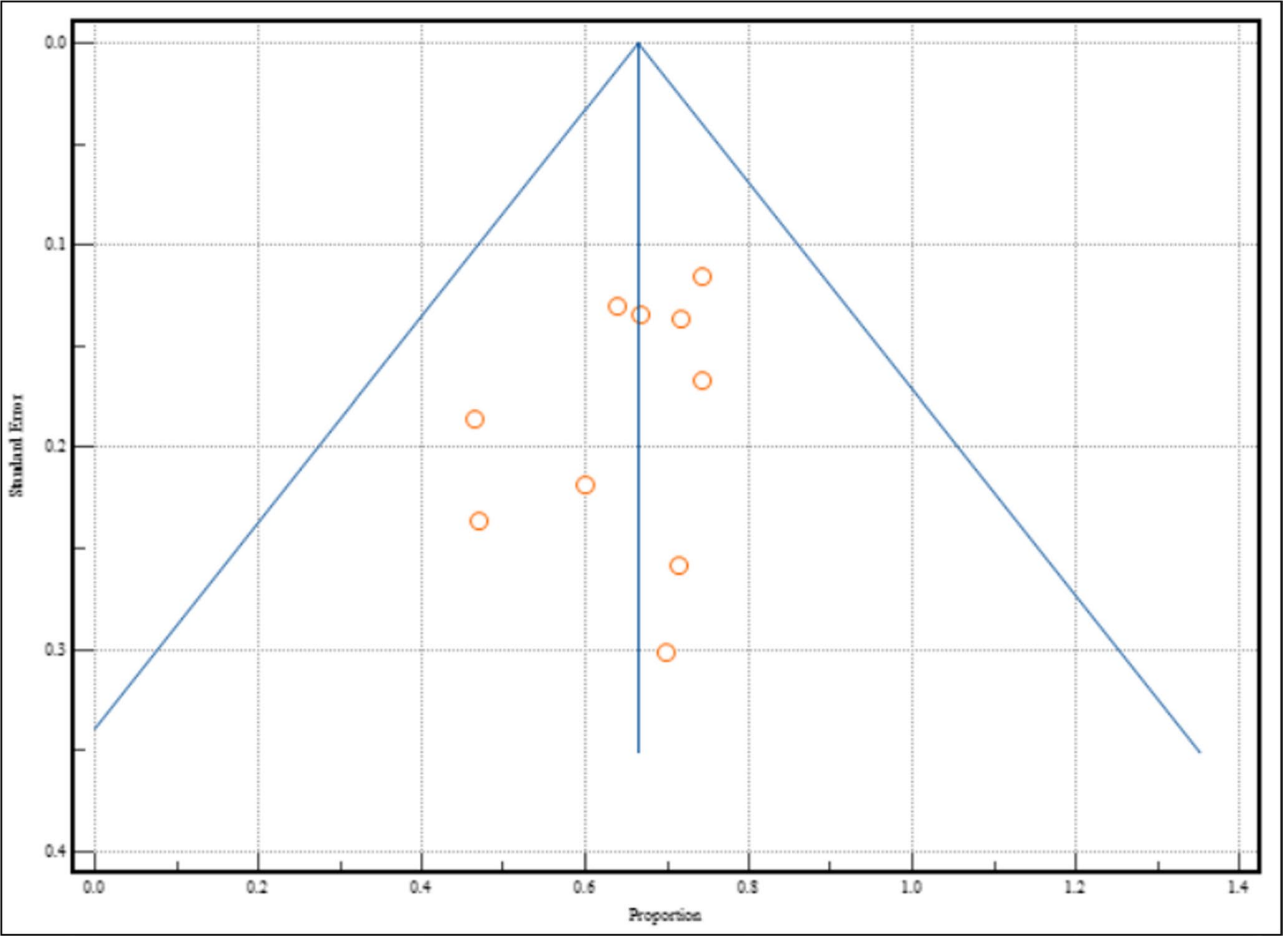
Funnel plot to assess publication bias

Critical appraisal of studies indicated an acceptably low risk of bias for all studies included. Potential sources of bias were most pronounced with participant selection, confounding, missing data, and post-exposure interventions in the non-randomized studies (Fig. 2). JBI assessment also found a low risk of bias for cross-sectional studies. All studies reporting on depression and anxiety prevalences were considered low risk across all risk bias appraisal tools, and a funnel plot with Egger’s test showed 10 of 10 studies fell inside the funnel plot with little asymmetry (−1.67 ([95% CI: −4.44-1.09], *p* = 0.201), suggesting low publication bias (Fig. 3) [20].

### Patient characteristics

A total of 401 patients with ENS were included with a mean age of 46.2 (range: 19–81; [95% CI: 42.8–49.6]); 59.5% were male [95% CI: 46.2–72.2]. The most common index surgery was turbinate surgery (75.1% [95% CI: 42.9–96.5]), and patients may have undergone additional nasal surgeries prior to the onset of symptoms. Following the index surgery, 38.4% [95% CI: 20.2–59.4] of patients were diagnosed with ENS within 3 years, 23.0% [95% CI: 8.9–43.5] between 3 and 10 years, and 42.2% [95% CI: 23.3–63.0] after 10 years. Among patients treated with augmentation, 10.0% [95% CI: 0.1–39.3] underwent injection and 90.0% [95% CI: 60.7–99.9] underwent implantation. Polyethylene was the most common material utilized (44.4% [95% CI: 3.4–91.5]). Most patients underwent bilateral implantation or injection (72.2% [95% CI: 63.3–80.9]). Pre-operatively, the mean score of ENS6Q was 20.8±1.1. Depression scores at baseline were 13.5±1.1 for PHQ-9 and 19.8±1.7 for BDI-II. Anxiety scores as baseline were 11.0±1.2 for GAD-7 and 20.2±1.6 for BAI. Further information on patient and treatment characteristics can be found in Table 2.

**Table 2:** Patient demographics

Demographics	
Sample Size (N)	401
Age (Mean, Range)	46.2 (42.8–49.6); range: 19–81
Male	59.5 (46.2–72.2)
Female	32.0 (27.4–37.0)
Body Mass Index (kg/m^2^)	24.3 (23.7–25.0)
Married	70.3 (55.7–82.4)
Unmarried	29.7 (17.6–44.3)
Pre-university education	68.1 (53.4–80.6)
University education	29.7 (17.6–44.3)
Post-graduate education	2.7 (0.2–11.8)
Smoking history	13.2 (5.9–22.7)
*Empty Nose Syndrome (ENS) Characteristics*	
Prior turbinate surgery	75.1 (42.9–96.5)
Prior septoplasty	63.5 (43.7–81.1)
Prior sinus surgery	22.9 (11.1–37.5)
Prior other nasal surgery	22.5 (9.7–38.6)
< 3 years since surgery	38.4 (20.2–59.4)
3–10 years since surgery	23.0 (8.9–43.5)
> 10 years since surgery	42.2 (23.3–63.0)
Preoperative Patient-Related Outcome Measures (PROMs)*	
Empty Nose Syndrome 6-Item Questionnaire (ENS6Q)	20.8 ± 1.1
Sino-Nasal Outcome Test-22 (SNOT-22)	54.1 ± 5.5
Sino-Nasal Outcome Test-25 (SNOT-25)	67.3 ± 2.6
Patient Health Questionnaire-9 (PHQ-9)	13.5 ± 1.1
Beck Depression Inventory-II (BDI-II)	19.8 ± 1.7
Generalized Anxiety Disorder-7 (GAD-7)	11.0 ± 1.2
Beck Anxiety Inventory (BAI)	20.2 ± 1.6
Patient Health Questionnaire-15 (PHQ-15)	11.1 ± 0.9
*Treatment Characteristics*	
Chinese traditional medicine (herbal-based, acupuncture, diet)	11.5 (4.2–23.7)
Carboxymethylcellulose/glycerin injection	10.0 (0.1–39.3)
Polyethylene implantation	44.4 (3.4–91.5)
Bone implantation	16.4 (0.02–53.7)
Small intestinal submucosa implantation	2.2 (0.1–7.2)
Dermal matrix implantation	4.9 (0.03–17.3)
Unilateral injection/implantation	27.3 (19.1–36.7)
Bilateral injection/implantation	72.7 (63.3–80.9)

*All values reported as proportion (%) with 95% confidence intervals*

*Baseline PROMs reported as mean and standard deviation

### Prevalence of depression and anxiety

The prevalence of depression among patients with ENS was 76.6% [95% CI: 61.0-89.1], and 61.4% [95% CI: 47.8–74.2] of patients suffered from moderate to severe depression (Table 3). In two studies comparing depression in patients with ENS to those with CRS, patients with ENS had a significantly increased rates of depression compared patients with CRS (RR: 2.8 [95% CI: 1.5–5.2], *p* = 0.001), and prevalence of moderate to severe depression was also increased (RR: 5.5 [95% CI: 3.1–9.7, *p <* 0.001). The prevalence of anxiety among patients with ENS was 77.0% [95% CI: 49.9–95.5]. Among patients with ENS, 40.2% [95% CI: 30.6–50.2] suffered from severe anxiety and 26.7% [95% CI: 15.4–39.8] suffered from moderate anxiety (Table 3).

**Table 3:** Prevalence of depression and anxiety

	Empty Nose Syndrome (ENS) (*n* = 82)	Chronic Rhinosinusitis (CRS) (*n* = 94)
*Depression prevalence*	76.6 (61.0-89.1)	24.8 (12.6–39.5)
No depression	22.8 (8.3–41.8)	75.2 (60.5–87.4)
Mild Depression	15.9 (11.5–21.2)	14.3 (8.0-22.9)
Moderate Depression	22.6 (13.1–33.8)	
Severe Depression	38.1 (26.6–50.3)	
Moderate - Severe Depression	61.4 (47.8–74.2)	12.4 (6.5–20.7)
*Anxiety prevalence*	77.0 (49.9–95.5)	
No anxiety	14.4 (4.8–27.9)	
Mild anxiety	18.7 (13.7–24.7)	
Moderate anxiety	26.7 (15.4–39.8)	
Severe anxiety	40.2 (30.6–50.2)	

*Prevalences reported as proportions (%) with 95% confidence intervals. Moderate to severe depression was reported as a single data point for CRS studies

### Augmentation treatment outcomes

**Fig. 4:**
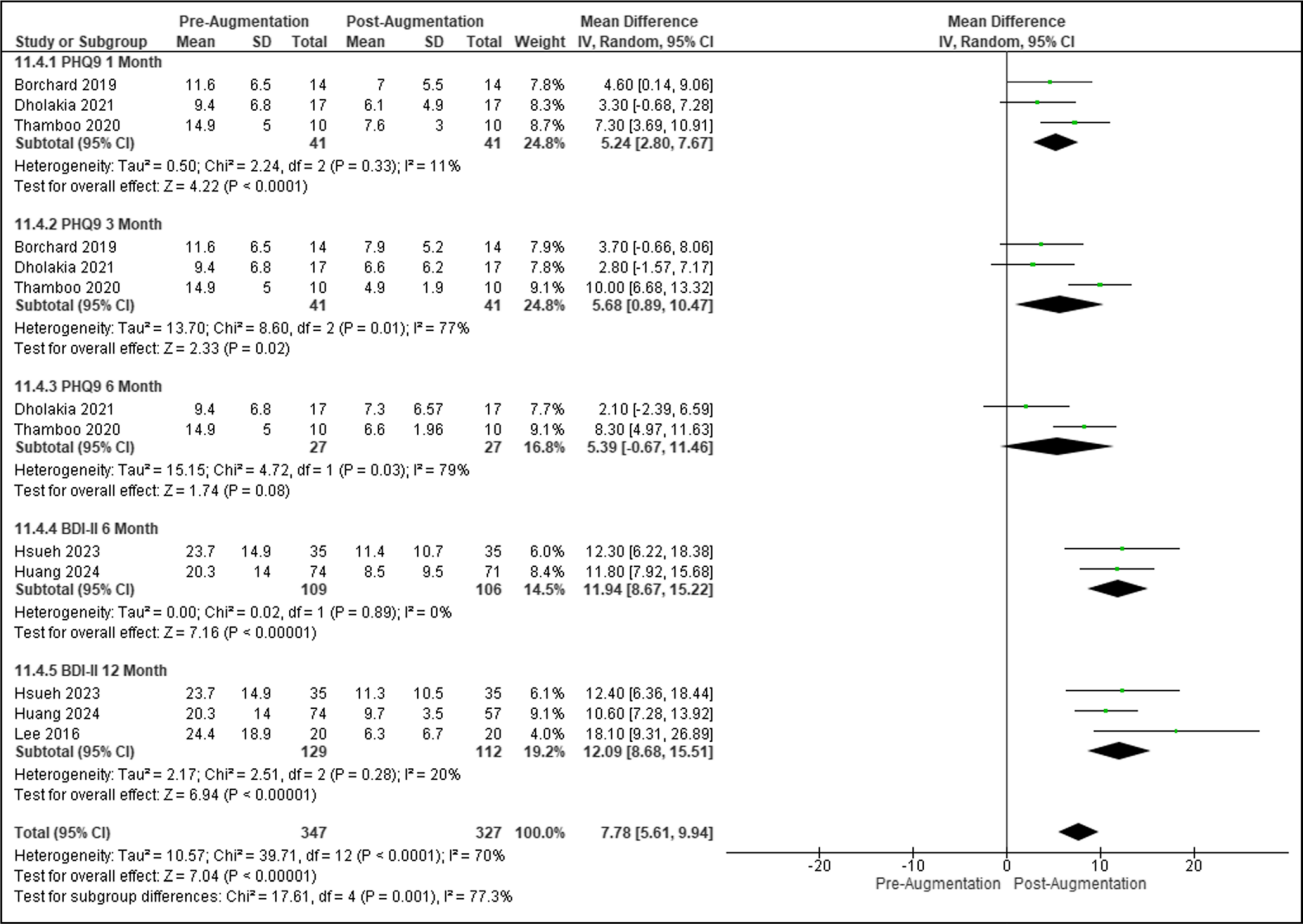
Forest plot of depression outcomes following augmentation

Standardized mean differences for depression (PHQ-9, BDI-II) from pre-augmentation to 1-, 3-, 6-, and 12-months following augmentation (injection or implantation) were 0.89 ([95% CI: 0.3–1.5], *p* = 0.004), 0.96 ([95% CI: 0.3–1.6], *p* = 0.003), 0.95 ([95% CI: 0.5–1.4], *p <* 0.0001), and 0.93 ([95% CI: 0.7–1.2], *p* < 0.00001) (Figure S5). When utilizing PHQ-9, mean differences for depression scores at 1-, 3-, and 6-months post-augmentation were 5.26 ([95% CI: 3.0-7.6], *p* < 0.00001), 5.68 ([95% CI: 0.9–10.5], *p* = 0.02), and 5.39 ([95% CI: −0.7-11.5], *p* = 0.08). When utilizing BDI-II, mean differences for depression scores at 6- and 12-months post-augmentation were 11.94 ([95% CI: 8.7–15.2], *p* < 0.00001) and 11.72 ([95% CI: 9.0-14.5], *p* < 0.0001) (Fig. 4 and S6).

**Fig. 5:**
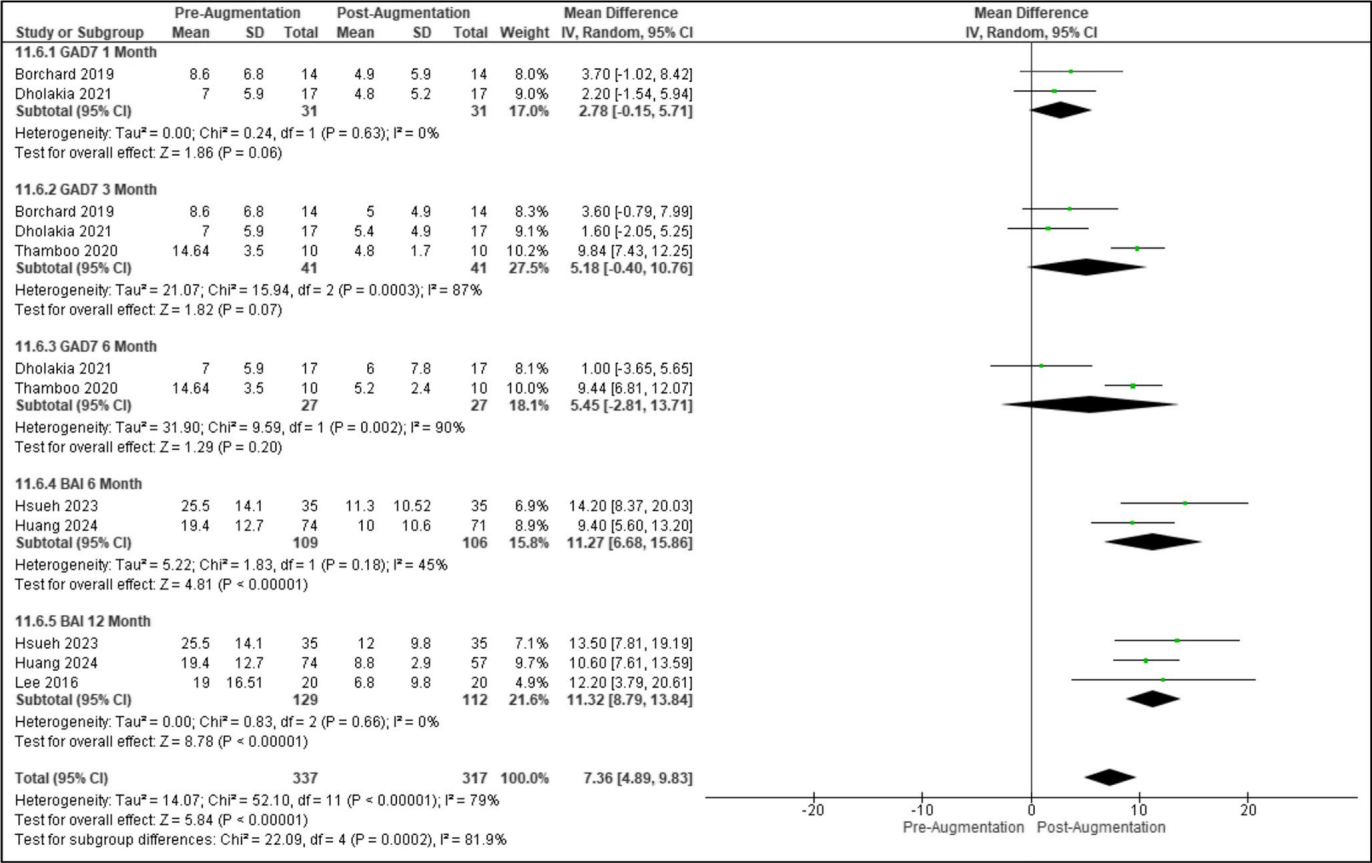
Forest plot of anxiety outcomes following augmentation

Standardized mean differences for anxiety (GAD-7, BAI) from pre-augmentation to 1-, 3-, 6-, and 12-months following augmentation were 0.47 ([95% CI: −0.04-1.0], *p* = 0.07), 1.08 ([95% CI: 0.2–1.9], *p* = 0.01), 1.04 ([95% CI: 0.4–1.7], *p* = 0.003), and 0.96 ([95% CI: 0.7–1.2], *p* < 0.00001) (Figure S5). When utilizing GAD-7, mean differences for anxiety scores at 1-, 3-, and 6-months post-augmentation were 2.78 ([95% CI: −0.2-5.7], *p* = 0.06), 5.18 ([95% CI: −0.4-10.8], *p* = 0.07), and 5.45 ([95% CI: −2.8-13.7], *p* = 0.20) post-augmentation. When utilizing BAI, mean differences for anxiety scores at 6- and 12-months post-augmentation were 10.83 ([95% CI: 7.7–14.], *p* < 0.0001) and 11.32 ([95% CI: 88.8–13.8], *p* < 0.0001) post-augmentation (Figs. 5 and S6).

Further information regarding SNOT-22, SNOT-25, and ENS6Q scores pre- and post-augmentation can be found in the supplement (Figure S3, S4, and S5).

## Discussion

In this study, over three-fourths of patients with ENS suffered from depression and anxiety, which is remarkably higher than that of the general population (8.3% for depression and 18.2% for anxiety) [29, 30]. The majority of patients experienced moderate to severe depression and anxiety manifesting as chronic fatigue, inability to concentrate, irritability, and even suicidal ideation, and are often driven by the persistent sensation of dyspnea and paradoxical nasal obstruction despite a patent airway [31]. These psychiatric comorbidities are not only common but also clinically significant, as they can exacerbate the subjective burden of ENS and negatively impact quality of life. The presence of depression and anxiety in ENS is not only a marker of disease burden but also a predictor of poorer surgical outcomes. Even after successful surgical intervention, a substantial proportion of patients continue to experience significant psychological distress, underscoring the need for a multimodal treatment approach [8, 9].

This study identified an increased risk of depression in ENS to CRS patients; however, only two studies compared depression in the ENS to CRS cohort, and neither addressed possible confounders [25, 26]. Thus, it remains unclear whether the risk of depression in ENS exceeds that of other sinonasal conditions, such as CRS. Schlosser et al. reported a prevalence of depression in CRS of 24.4%, consistent with the prevalence of depression of 24.8% in the CRS cohort included in our study [32–35]. Compared to the general population, rates of depression are nearly three times higher in CRS and over nine times higher in ENS, and the risk of depression in ENS compared to CRS remains to be confirmed.

Although psychiatric symptoms demonstrated improvement following augmentation, outcomes were inconsistent depending on the specific PROM utilized. PHQ-9 scores showed significant improvement at 1- and 3-months, but not at 6-months. GAD-7 scores did not reveal significant improvement at any time point post-augmentation. BDI-II and BAI demonstrated significant improvement at 6- and 12-months post-augmentation. Effect sizes of PHQ-9 and GAD-7 barely surpassed their respective minimal clinically important differences (MCID), while those of BDI-II and BAI surpassed theirs to a larger degree [36–38]. This discrepancy in PROM-dependent outcomes might be attributable to the fact that the PHQ-9 has a lower sensitivity and higher specificity than BDI-II [39, 40]. In terms of the content of the PROMs, the PHQ-9 is shorter, freely available, and aligns with diagnostic criteria, while the BDI-II is better suited to capture different dimensions of depression severity [41–43]. As demonstrated by this study, current mental health PROMs used to assess psychiatric status in ENS are not adequately capturing the impact of augmentation on depression and anxiety, underscoring the need to develop a tool designed to address the nuances of mental health in patients with ENS.

In a study by Li et al., computational fluid dynamics analysis in patients with ENS revealed disrupted nasal aerodynamics and impaired sensorineural sensitivity [44]. The disconnection between objectively normal anatomy and severity of symptoms reported by patients with ENS may be attributed to individual variability in mucosal sensitivity with a possible psychiatric component of hyperfixation on airflow perception [45, 46]. This subjectivity underscores a critical limitation in understanding why some patients develop ENS-like symptoms despite having similar anatomical changes to others who remain asymptomatic, leading to a lack of data on psychologic function prior to and following turbinate surgery. Furthermore, as seen among the included studies, ENS is diagnosed and monitored primarily on patient-reported symptoms using the ENS6Q and cotton test, thus introducing a strong subjective bias in the diagnostic criteria, and further emphasizing the necessity for standardized diagnostic criteria in the evaluation of ENS (Table 1) [47].

Given that the condition of ENS is diagnosed based upon a subjective basis, it is possible that relief in symptoms following rhinologic treatment may be subject to similar placebo effects. Although sham surgery has yet to be investigated in patients with ENS, sham procedures for other rhinologic conditions have demonstrated a relative PROM improvement of 48.4% in rhinologic symptom scores [48]. Additionally, somatization has been reported as a condition associated with ENS [49]. Lamb et al. reported that patients with ENS had a significantly increased risk of Somatic Symptom Disorder as measured by PHQ-15 compared to patients with CRS (53.1% vs. 14.0%, *p* < 0.0001) [26]. Similarly, Tian et al. found that 35.7%, 39.3%, 21.4% of patients with ENS were rated as having mild, moderate, and severe somatization disorder, respectively. Following treatment with antidepressants and/or cognitive behavioral therapy (CBT), mean scores in PHQ-15 as well as PHQ-9, GAD-7, and SNOT-25 improved significantly from pre-treatment to 12-months following treatment, suggesting a strong potential role for psychiatric intervention in the management of both rhinologic symptoms as well as depression and anxiety [12].

In our study, augmentation for ENS demonstrated an improvement of 17.2% in ENSQ6 scores. Symptoms tend to significantly improve in PROM scores in the early post-operative period, then demonstrate a “wearing-off” effect as PROM scores regress [50]. In our study, this can be seen with PHQ-9 scores, which diminish in significance from 1-month to 3-months then lose significance at 6-months following augmentation. Treatment of ENS with augmentation may result in differential improvement in a variety of specific symptoms. At 1-month post-augmentation, two studies report significant improvements in dryness, crusting, and diminished airflow sensation [21, 22]. At 3-months, two studies reported improvements in dryness and suffocation [8, 22]. Improvements in the symptom of ‘nose too open’ were inconsistent across studies. Borchard et al. (2019) and Huang et al. (2021) reported no improvements in this symptom 1- and 3-months, while Dholakia et al. (2021) reported significant improvement at 1- and 3-months, then no improvement at 6-months and 1-year post-augmentation, further demonstrating a potential “wearing off” effect [8, 21, 22].

A systematic review and meta-analysis by Hussain et al. found significant improvements in overall rhinologic symptom, anxiety, and depression scores during follow-up of up to 1 year, proposing that ENS management should center around surgical intervention and be supplemented with psychiatric and medical management [5]. Although surgical treatment options show promising results, the long-term efficacy and treatment non-inferiority has yet to be supported by adequately powered studies with control groups. Additionally, non-invasive treatment options catered to both therapeutic (CBT and nasal rinses/humidification) and pharmacologic (antidepressants and topical corticosteroids) have shown success in controlling psychological and rhinologic symptoms of ENS [6, 12]. Treatment outcomes of ENS are multifaceted based on factors such as patient subjectivity of airflow hyperfixation and/or mucosal hypersensitivity, possible somatization, potential wearing-off effect, and augmentation targeting some, but not all ENS symptoms. While augmentation has been shown to improve symptoms diminished airflow sensation, dryness, and crusting, symptoms based on mucosal sensitivity, such as pain and obstruction, may be more effectively targeted with psychological therapy. Additionally, mechanisms of ENS, such as scarring or mucosal atrophy, are hypothetical and remain an area for further study. Thus, comprehensive management is absolutely essential in the treatment of ENS, and optimal treatment may require augmentation procedure as well as early psychiatric intervention and involvement by mental health professionals.

This study faces several limitations. First, in this study, most patients presented with ENS symptoms ten or more years since the index surgery and not all studies excluded prior psychiatric co-morbidities, thus it is challenging to determine whether patients had pre-existing psychologic diagnoses prior the index surgery that led to the development ENS as well as the outcomes of concomitant psychiatric and surgical treatment, preventing any determination of causality (Table 1). Secondly, many studies included overlapping populations which may artificially inflate the sample size, and thus efforts were made to exclude duplicates by selecting for the most comprehensive data. Furthermore, to address potentially inadequately powered sample sizes, a leave-one-out test was conducted for each effect-size estimate. Thamboo et al. was excluded in the analysis for mean difference measured by GAD-7 at 1-month and standardized mean difference for anxiety at 1-month, but included for the remaining mean differences as removing it merely diminished but did not eliminate significance [28]. Thirdly, variability in patient populations, intervention protocols, and outcome measures contributed to substantial statistical heterogeneity (I² = 50%), which was addressed by adjusting statistical models with random effects. Future directions should assess if patients with underlying psychological co-morbidities may have a predisposition to the development of ENS following nasal surgery to clarify the nature of the relationship between mental health conditions and ENS to guide treatment, as well as investigate the role of CBT and psychiatric medications as adjunct treatments.

## Conclusion

The ENS patient population experiences very high prevalences of both depression and anxiety. Patient-reported depression and anxiety symptoms appear to improve after augmentation procedures, although there is variability across timepoints and specific instruments. Optimal treatment of ENS should likely address both rhinologic and psychiatric symptoms, with a potential role for early psychiatric intervention and involvement by mental health professionals given the high prevalence of both depression and anxiety in this patient population.

## Supplementary Information

Below is the link to the electronic supplementary material.

ESM 1 (DOCX 227 KB)

## Data Availability

Data can be accessed upon reasonable request to the corresponding author.
