# Supplementary material for: Depression and anxiety in empty nose syndrome: A systematic review and Meta-analysis
Source: Eur Arch Otorhinolaryngol. 2025 Jul 5;282(12):6037–48. doi: 10.1007/s00405-025-09535-1 (PMC12680857; doi:10.1007/s00405-025-09535-1)
Supplement: Supplementary file 1 — (DOCX 227 KB) [file 405_2025_9535_MOESM1_ESM.docx]

**Journal:** European Archives of Oto-Rhino-Laryngology

**Title:** Depression and Anxiety in Empty Nose Syndrome: A Systematic Review and Meta-analysis

**Authors:** Anuja H. Shah, BA^1,2^; Isabelle J. Chau, BS^1,3^; Shaun A. Nguyen, MD^1^; Alexander N. Duffy, MD^1^; Zachary M. Soler; MD^1^, Rodney J. Schlosser, MD^1^

1. Department of Otolaryngology – Head and Neck Surgery, Medical University of South Carolina, Charleston, SC, USA
2. Georgetown University School of Medicine, Washington, DC, USA
3. New York Medical College School of Medicine, Valhalla, New York, USA

**Corresponding Author:**

Shaun A. Nguyen, MD

MUSC Department of Otolaryngology – Head and Neck Surgery

135 Rutledge Ave, Charleston, SC 29425

[nguyensh@musc.edu](mailto:nguyensh@musc.edu)

**Supplementary Materials**

**S1.** Final Search Strategy

*This provides an overview of the search terms used to guide the systematic review of the literature.*

("empty nose*" OR "atrophic rhinitis*" OR "nasal obstruction*" OR "turbin*") AND ("psyc*" OR "neuropsyc*" OR "mental*" OR "anxi*" OR "depress*" OR "panic*" OR "stress*" OR "obsessive-compulsive*" OR "somatic symptom*")

- 1. 822 results in PubMed (8/5/24)- filters: human
  2. 478 results in Scopus (8/5/24) - filters: human
  3. 111 results in CINAHL (8/5/24) - filters: human
  4. 71 results in PsychINFO (8/5/24) - no restrictions
  5. 13 results in Central (8/5/24) - no restrictions

**S2.** JBI Cross-Sectional Risk of Bias Assessment

*This chart displays the sources of bias among the cross-sectional studies included*


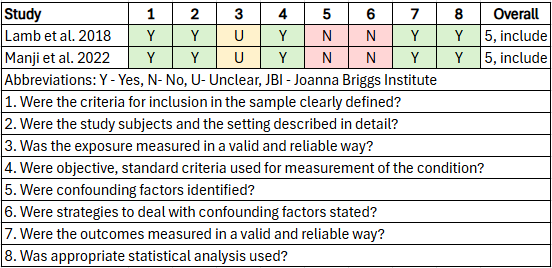


*Abbreviations: Y - Yes, N- No, U- Unclear, JBI - Joanna Briggs Institute*

**S3**. Empty Nose Syndrome 6-Item Questionnaire Mean Differences

*This graph displays the mean differences Empty Nose Syndrome 6-Item Questionnaire scores from pre-treatment to post-treatment time points among the studies included.*


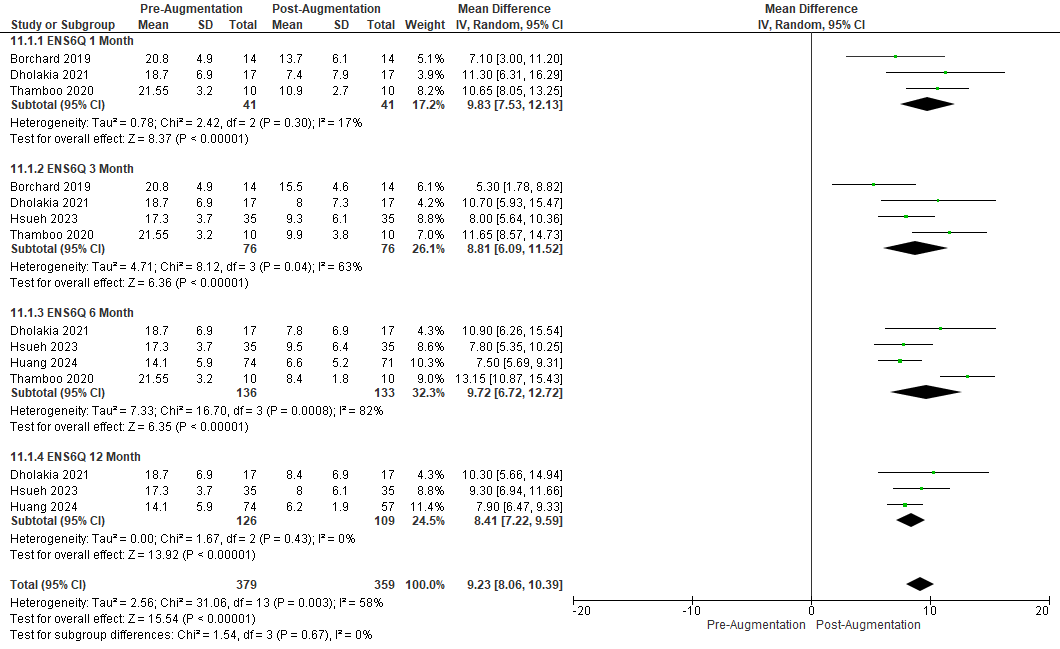


**S4**. Sino-Nasal Outcome Test-22 and -25 Mean Differences

*This graph displays the mean differences Sino-Nasal Outcome Test-22 and -25 scores from pre-treatment to post-treatment time points among the studies included.*


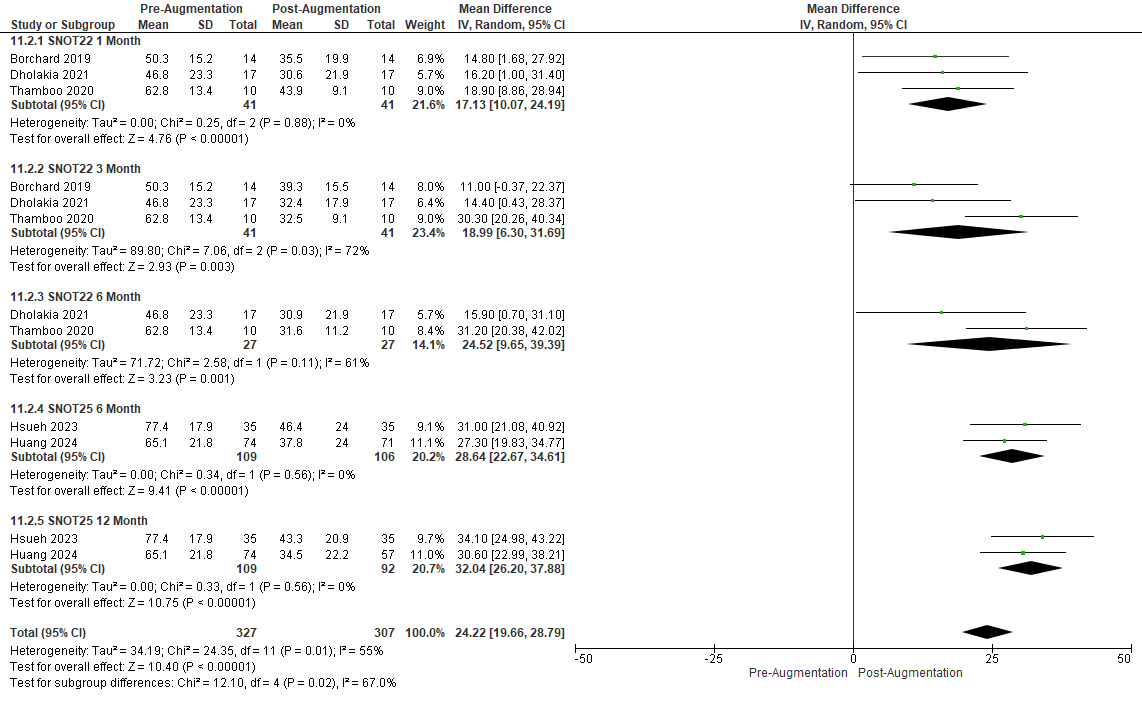


**S5**. Standardized Mean Differences for Rhinologic Symptoms, Depression, and Anxiety

*This graph displays the standardized mean differences for all patient-related outcome measures reporting on rhinologic symptoms, depression, and anxiety from pre-treatment to post-treatment time points among the studies included.*


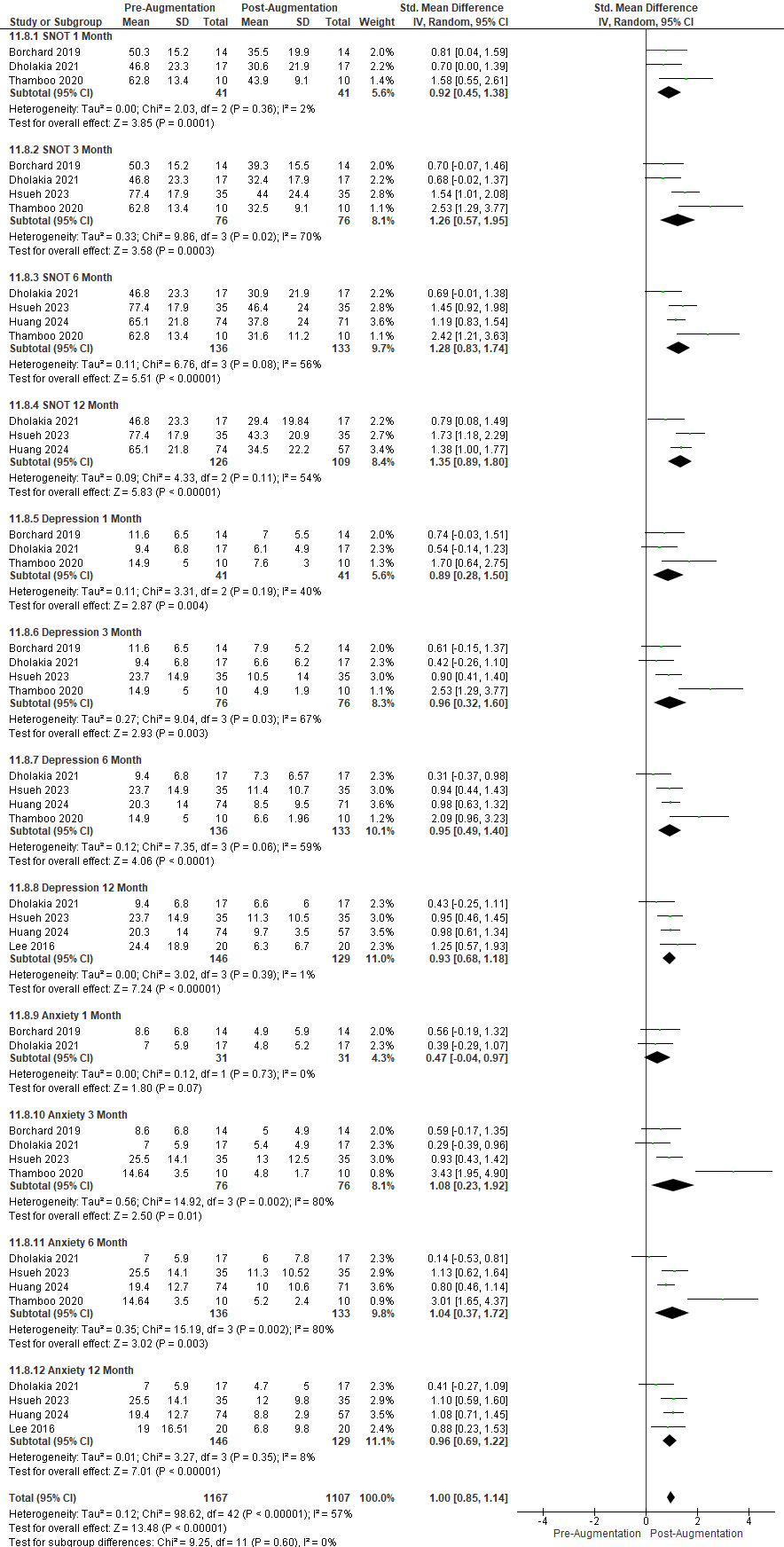


**S6.** Patient-Related Outcome Measures Over Time


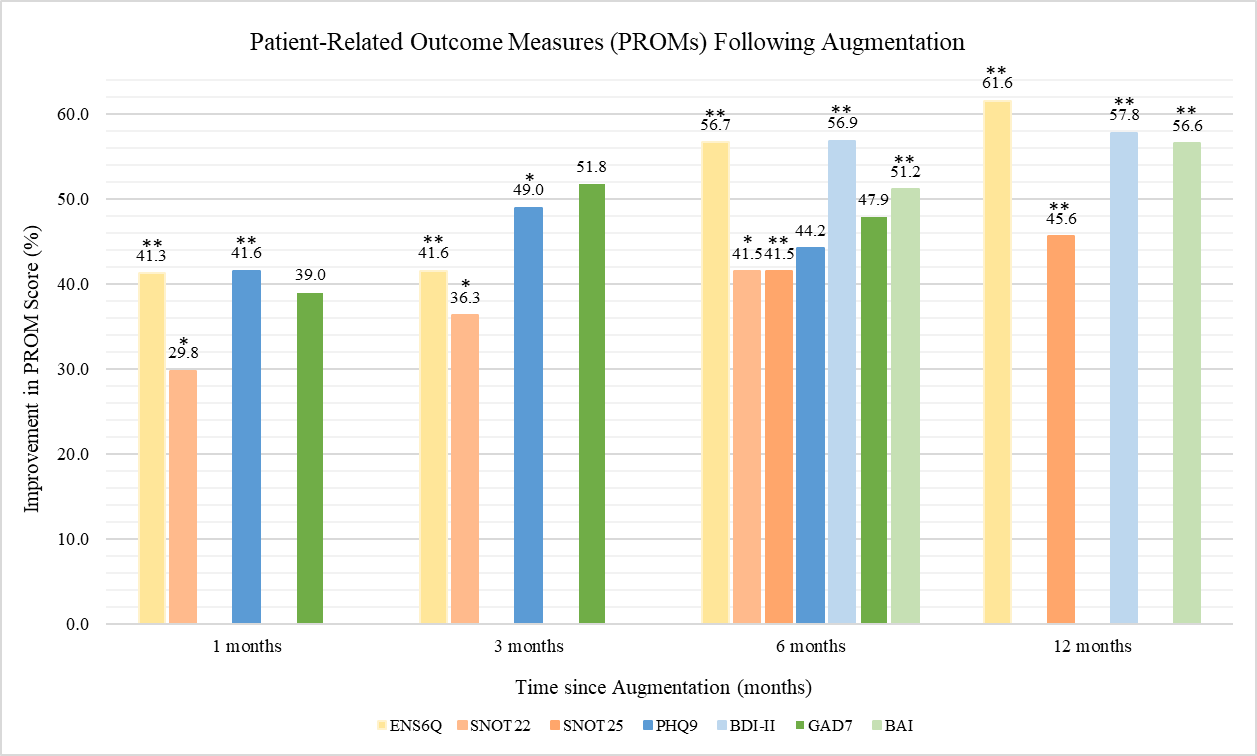


** denotes p<0.05 significance*

*** denotes p<0.0001 significance*
